# Selections and Abstracts

**Published:** 1912-03

**Authors:** 


### SELECTIONS AND ABSTRACTS

### SURGICAL SUGGESTIONS.

A breaking-down sarcoma of the ilium may simulate a gluteal aneurism.

Not infrequently subacute inflammations of the Fallopian tubes will be found to be of tuberculous origin.

A mild degree of shock causes an increase in leucocytes; severe shock paralyzes the leucoblastic function.

A pulsating tumor in the side may be an aneurism of the abdominal aorta although palpation fails to disclose its connection with the aorta.

Before performing esophagotomy for foreign body, make a final examination (radiographic or otherwise) to determine that the object has not slipped into the stomach.

Persistent dyspareunia, with no other ascertainable cause, may often be found to lie in a chronic gonorrhea of Skene's ducts.

Some apparently inoperable carcinomata of the cervix will yield remarkably to repeated cauterization with the actual cautery and zinc chloride.

Any menstrual irregularity or abnormality in a women who has hitherto been perfectly regular and normal in her menstual periods, should always suggest the possibility of an ectopic pregnancy.

Injection into a fistula in ano of a staining solution or a colored paste makes it possible for the operator to assure himself that all branches of the tract have been explored.

It is worth while inaugurating the treatment of "idiopathic" pruritus ani by the administration of santonin or of enemata of quassia infusion—seat-worms may escape discovery on one or two examinations.

If skiagraphs show the shadow of a calculus at the neck of the bladder when the patient is exposed lying flat with the organ empty, and in the same position when the pelvis is elevated and the bladder full, the stone is in the prostate (or prostatic urethra) or in a diverticuum behind the prostate.—American Journal of Surgery.

---

### PAPYRUS EBERS.

Some time ago, announcement was made through the pages of this journal that Dr. Carl H. von Klein had completed an English translation of the Papyrus Ebers, and that its publication in book form depended upon an advance subscription list of one thousand names. As a result of that announcement and the interest shown by Dr. von Klein's friends, six hundred subscriptions have been secured. The enterprise drags at this point and something must be done to arouse the profession to the importance of preserving this valuable manuscript. Four hundred additional subscriptions must be secured.

The Editor has had an opportunity to examine the elaborate manuscript, which is preserved with the greatest care and affection by the venerable translator, and one realizes how important it is that the author of the translation should see the ideal work of his life through the press. It is a quesion, in fact, whether the translation will not be lost to science just as Ebers' work was lost to us, by the author taking it with him to his bier, if he is not spared to finish the work of publication.

Dr. von Klein has spent twenty years of odd moments of a busy, useful life, mostly devoted to the literature of our profession, on this work of love, and it will be regrettable if it is now lost when so little will not only preserve it to us, the profession, but give its faithful author the satisfaction of seeing it in print.

The book will consist of 650 pages, 7 x 10 inches, in two colors (red and black) similar to the original, with six plates, bound in one volume. Subscriptions may be sent directly to Dr. Carl H. von Klein, Medical Department, John Crerar Library, Chicago, Illinois. Dr. von Klein will publish this volume personally, and it will be sold only on subscription.—Editorial Surgery, Gynecology and Obstetrics, January, 1912, page 94.

